# Home, school, and the hidden cost of parental mental health

**DOI:** 10.3389/fpsyg.2025.1643927

**Published:** 2026-02-16

**Authors:** Jingwen Zhou, Stephanie Del Tufo

**Affiliations:** 1 Human Development and Family Sciences, University of Delaware, Newark, DE, United States; 2 School of Education, University of Delaware, Newark, DE, United States; 3 Interdisciplinary Neuroscience Graduate Program, University of Delaware, Newark, DE, United States; 4 Department of Psychological and Brain Sciences, University of Delaware, Newark, DE, United States

**Keywords:** parental mental health, psychosocial determinants, childhood socioeconomic status, school socioeconomic status, family socioeconomic status, home socioeconomic status, childhood inequality, developmental inequality

## Abstract

This study examines the association between parental mental health and children’s socioeconomic status (SES) across both home and school environments, using nationally representative data from the Adolescent Brain Cognitive Development (ABCD) Study. Traditional childhood SES markers often focus on parental income, occupational prestige, and maternal education; however, they may not fully capture how children’s proximal experiences of SES differ across daily contexts due to parents’ personal challenges. Employing latent variable path analyses (LVPA), we explored whether parents’ mental health, often a critical aspect of childhood adversity, shapes both home-based and school-based SES. Our findings revealed that poorer parental mental health is significantly linked to more disadvantaged SES in home and school settings. This suggests that parental mental health may affect not only the resources families possess but also the degree to which children benefit from those resources across critical developmental settings. These findings highlight the importance of addressing parental mental health as a key mechanism in understanding and reducing invisible developmental inequality.

## Introduction

Children are strongly influenced by their surrounding environments, especially by an immediate one: the family. Socioeconomic status (SES) is one of the most significant family characteristics that impacts a child’s human capital accumulation, including educational and health outcomes. As core dimensions of SES, most studies have examined the relation between childhood SES and child outcomes using family-based measures such as parental education, occupation, and/or income. However, childhood SES is a complex concept. The 2012 National Assessment of Educational Progress (NAEP) guideline titled *Improving the Measurement of Socioeconomic Status for the National Assessment of Educational Progress: A Theoretical Foundation* proposed that SES should be evaluated using a contextually diverse approach. This approach accounts for diverse developmental settings beyond home, including school and neighborhood environments (Cowan et al., 2012). As such, this study aims to determine the association between focal measures of childhood SES and a well-established type of childhood adversity: parental mental illnesses.

Mental illness is currently among the most prevalent health conditions in the United States (Centers for Disease Control and Prevention (CDC), 2023). A significant adult population is affected, with over one in five individuals living with a mental illness (Centers for Disease Control and Prevention (CDC), 2023). Nearly one in every 20 U.S. adults is living with a severe mental health illness, such as schizophrenia, bipolar disorder, and major depression (Substance Abuse and Mental Health Services Administration [SAMHSA], 2022). Worldwide, around one billion people are affected by mental illness (World Health Organization, 2025). Prior to the COVID-19 pandemic, the US, Canada, and Sweden were among the countries reporting the highest likelihood of being diagnosed with depression, anxiety, or other mental health conditions by a doctor (Tikkanen et al., 2020).

Considering these alarming statistics, it is likely that mental illnesses affect not just the individuals diagnosed but also demonstrate ripple effects to those in their care. Over one in 14 children in the U.S. are in the care of an adult struggling with a mental health difficulty (Wolicki et al., 2021). One in 4 children are in the care of a parent with a mental illness in Australia (Maybery et al., 2009, 2019) and in the United Kingdom (Abel et al., 2019). These high prevalences highlight that parental mental illness is affecting a substantial proportion of children worldwide. This underscores the significance of examining how parental mental health influences children, including the pathway through which it shapes the economic and social resources available to children.

In addition to prevalence statistics, several conceptual models call for greater attention to parental mental health as a critical factor in children’s development. Developmental psychopathology models, such as Goodman and Gotlib’s (1999) Model of Intergenerational Transmission of Risk for Depression, illustrate how maternal depression impacts children both neurobiologically and environmentally, leading to adverse child outcomes. Building on this, Hosman et al. (2009) expand the scope to include fathers and a broader range of mental health disorders. Across these models, parental mental health impacts children through mechanisms such as neurobiological vulnerability, disrupted parenting, heightened exposure to stress, and poverty (Goodman and Gotlib, 1999; Hosman et al., 2009). These insights provide a conceptual foundation that motivates the present study’s examination of how parental mental illness is linked to children’s socioeconomic conditions. While socioeconomic conditions are not a child outcome per se, they represent a critical pathway through which developmental opportunities are either limited or facilitated.

As such, this study aims to has a dual focus: (1) determining how parental mental health is associated with childhood SES within two-parent families, and (2) elucidating what differences, if any, exist when considering childhood SES across home and school environments.

### Theoretical frameworks

Understanding how parental mental illness relates to children’s socioeconomic conditions across daily environments requires an integration of complementary theoretical tools. In the present study, we draw on three distinct but interconnected frameworks that collectively capture the complexity of this association: (1) the previously discussed intergenerational models of psychopathology through which parental mental illness influences child outcomes; (2) a mapping system that differentiates multiple developmental contexts of children within which these processes unfold; and (3) a sociological perspective that focuses on the interplay between health inequalities and socioeconomic stratification.

#### Urie Bronfenbrenner’s ecological systems theory

Following the intergenerational models of psychopathology (Goodman and Gotlib, 1999; Hosman et al., 2009), we draw on Bronfenbrenner’s Ecological Systems Theory (Bronfenbrenner, 1979, 1994) to situate the influence of parental mental health within children’s immediate developmental environments (e.g., home and school), where socioeconomic conditions are experienced. Bronfenbrenner (1979, 1994) posited that a developing child is surrounded and affected by multilayered systems in society. He argued that while children are influenced by distal factors, such as social norms, culture, and public policies, they are most directly impacted by their family environment. Guided by EST, it is possible to consider how various interconnected systems might shape children’s development. For instance, parents’ mental health, a significant concern within the family environment, has been widely reported to impact children across diverse empirical research (e.g., Hosman et al., 2009; Kamis, 2020). This is because parental mental health does not exist in isolation but interacts with other factors such as family income stability, income-to-needs ratios, residential areas, and children’s schooling choices (Jabbari et al., 2022; Scrimin et al., 2022).

However, in addition to the immediate home environment in the microsystem, the EST framework also emphasized the significance of proximal environments, such as school and neighborhood, in shaping a developing child. Beyond the home environment, schools also represent a crucial component in the microsystem that directly impacts the child. In schools with high ratios of economically disadvantaged students, more challenges emerge. These schools often face issues such as fewer resources, larger class sizes, and greater barriers to parental engagement, which can affect the quality of education (Borg et al., 2012; McGee, 2021). Such school environments can create additional stressors for children that may amplify the influence of their home environments.

It is important to note that while in many contexts residential location determines school attendance, the direct home-school linkage can be weakened among families affected by parental mental illness. Thus, by examining these interconnected factors within Bronfenbrenner’s EST, we can gain a comprehensive understanding of how home environments and broader societal structures shape children’s SES trajectories.

#### Fundamental cause framework

The Fundamental Cause Framework (FCF) (Hayward and Gorman, 2004) posits that adverse early life conditions, stemming from socioeconomic disparities, can set off a cascade of negative socioeconomic events (e.g., distressed lifestyle, work environment, and income trajectories), culminating in long-term effects on individuals’ well-being. Parental mental illness is considered one of these adverse early life conditions, significantly impacting the family’s SES and the quality of the environment in which a child develops. Poor mental health in parents not only leads to unstable employment, reduced educational attainment, and lower family income (Cornaglia et al., 2015; Luciano and Meara, 2014), but also adversely affects the child’s SES at home and their early SES-related behaviors in school (Kamis, 2020; Karim et al., 2022). These childhood emerging properties set the foundation for individuals’ later SES in adulthood. In the context of parental mental health, the FCF provides a tool to understand how disparities in mental health can lead to persistent socioeconomic inequities across generations.

### Development of proximal measurements of socioeconomic status

Socioeconomic status (SES) is a complex, multifaceted socio-demographic factor used across various disciplines. In adults, SES is typically measured using indicators such as educational attainment, occupational status, and income (Antonoplis, 2023; Cowan et al., 2012; Long and Renbarger, 2023). These proxies are sometimes referred to as the “big three” (Willms and Tramonte, 2015). In children, SES is measured using a greater variety of proximal factors that often include “access to financial, social, cultural, and human capital resources” (Cowan et al., 2012). However, most studies on children only consider SES proxies that have been previously reported in adult studies (e.g., educational attainment, occupational status). This has resulted in a dominant, albeit limited, view of childhood socioeconomic status, which does not allow for multidimensional components of childhood SES to be examined or compared.

While many measures of SES have been suggested over the last 100 years, the two most prominent are the Four-Factor Index of Socioeconomic Status (Hollingshead, 1975) and the Occupational Prestige Score (Nakao and Treas, 1992). Hollingshead’s (1975) “Four-Factor Index of Socioeconomic Status” is perhaps the most widely recognized measure of SES. Hollingshead’s index contains four domains based on the U.S. 1963, 1964, and 1970 Census classifications: marital status, retired/employed status, educational attainment, and occupational prestige. Despite Census data that is over 50 years old, this comprehensive index for assessing SES has been widely adopted and used in studies across fields (Adams and Weakliem, 2011; Edwards-Hewitt and Gray, 1995).

Nakao and Treas (1992) developed the 1989 Index of Socio-Economic Index of Occupations, which made a significant contribution to the field of SES measurement due to the specific focus on occupation status. This landmark index was formulated based on a major national inquiry into occupational prestige conducted in 1989 by the National Opinion Research Center (NORC) at the University of Chicago. They first identified 503 detailed occupational categories, assigning each an estimated prestige score. Each prestige score was then regressed on education and income to reveal the association of these two occupation-related factors with occupation prestige. Nakao and Treas (1992) found that both education and income are significant in determining the prestige of various occupations, with education playing a more substantial role. This approach not only improved the accuracy of occupational prestige rankings but also established a framework for SES measurement. This model has been widely adopted by SES researchers (e.g., Hauser and Warren, 1997; Song and Xie, 2023) for updating ranking systems over time as new occupations emerge.

Over the last two decades, childhood SES measure has often been measured using one or two of the three proxy indicators discussed above. For example, studies leave out parental occupation and include only parents’ education and family income (e.g., Fujishiro et al., 2010). Alternatively, studies often use only parental educational attainment (or maternal educational attainment), leaving out both family income and parental occupation (e.g., Benavente-Fernández et al., 2019; Justice et al., 2020). The omission of one or two indicators may be due to data availability or strategic considerations. Consequently, differences in how childhood SES is operationalized may account for inconsistent findings in this area of research.

Previous approaches that rely on only one or two proximal SES indicators are increasingly being challenged. For example, the National Assessment of Educational Progress (NAEP), administered by the National Center for Education Statistics (NCES) to representative samples of U.S. students in grades 4, 8, and 12. Over time, the NAEP program has frequently updated its SES measures. In 2012, a panel of leading SES scholars, including Hauser and Cowan, proposed a significant update to conceptual framework for measuring SES. The panel recommended defining childhood SES as a composite of parental educational attainment, occupation, and household income, which is conventionally referred to as home SES (Cowan et al., 2012). Following this change, a childhood SES construct containing all three proximal indicators has gained traction across disciplines. For instance, studies have examined the association of this SES on children’s self-esteem (Khundrakpam et al., 2020) and psychological adjustment (Karim et al., 2022).

#### Revisiting childhood SES

In our review of the literature, we found that “childhood SES” is a widely used term across disciplines (e.g., Cohen et al., 2010; Cowan et al., 2012; Ensminger and Fothrgill, 2014; Howe et al., 2012; Webb et al., 2017), indicating broad recognition of its importance. However, despite this widespread usage, there is no universally accepted definition for childhood SES. One possible reason for the lack of a universally agreed-upon measure of childhood SES may be that, while the home environment is highly correlated with SES, it does not fully determine the external environment, as previously discussed in the EST model (Bronfenbrenner, 1979, 1994).

This difficulty in reaching a consensus stems from varying views on what constitutes SES in childhood and how to most accurately measure it (Webb et al., 2017). Researchers argue over whether childhood SES should encompass not only financial factors such as household income and parental occupation but also exposure to childhood economic difficulties before the age of 16 (Laaksonen et al., 2005), childhood housing conditions (Howe et al., 2012), school characteristics (Cowan et al., 2012) and neighborhood characteristics (Webb et al., 2017). These perspectives have made it difficult to establish a universally accepted measure that captures the complexity of SES during childhood. This lack of a standard definition has drawn attention from international bodies. The Organisation for Economic Co-operation and Development (OECD), through its Program for International Student Assessment (PISA), has called for the development of an SES measurement for school-age children that captures the essential aspects of SES and child-related outcomes (Avvisati and Wuyts, 2024).

Given the absence of a standard definition, researchers often rely on available or conventional variables to define childhood SES and sometimes redefine the concept of SES by adding new variables and giving the resulting construct a new name. For example, in a study conducted by Yang et al. (2017), early-life SES and adult physiological functioning were examined, with early-life SES measured by parental education, household income, welfare receipt, and subjective financial well-being. Similarly, the Common Cold Project at Carnegie Mellon University measured childhood SES by including the mother’s and father’s respective educational attainment and whether parents owned or rented their home (Carnegie Mellon University, 2024). The socioeconomic backgrounds of children are complex as they are constituted by an interaction of various aspects and settings of their daily lives (e.g., Cowan et al., 2012).

Although an extensive body of research links childhood SES to developmental outcomes (e.g., Strickhouser and Sutin, 2019; Kininmonth et al., 2020; Takeuchi et al., 2021) much of this work equates childhood SES with home SES, overlooking the broader environments and experiences that shape a child’s socioeconomic circumstances.

#### Emerging SES in school

A closely related discussion is that children’s emerging SES (Cohen et al., 1997) is influenced by their unique childhood experiences (e.g., neighborhood safety, peer interactions) and individual differences (e.g., executive functions). Notably, children’s emerging SES can diverge from the SES indicators conventionally associated with their parents (Hu et al., 2024; Wei et al., 2021). These emerging SES indicators could involve their neighborhood characteristics, school contexts, social interactions, and other factors (Kohen et al., 2008; Opara et al., 2023; Smart et al., 2019), all of which contribute to children’s immediate socioeconomic environment and overall development. While families influence some of these factors, particularly neighborhood and school selection, the child’s lived experience within these environments is shaped by interactions and circumstances beyond parental control.

Empirical studies have examined how children’s experiences and environments shape their emerging SES, independent of parental SES. For example, Keys et al. (2013) explored how preschool center quality, which is often associated with school SES, impacted children’s language and mathematics outcomes. Their findings indicated significant positive associations between center quality and children’s readiness, with little evidence of moderation by family SES. Likewise, Entwisle et al. (1994) investigated potential neighborhood origins of variation in boys’ mathematics performance. They found that school-age boys’ mathematical reasoning skills were related to the type of neighborhood they lived in. Entwisle et al. (1994) speculating that these differences might stem from varying opportunities to engage in complex rule-based games with peers (Entwisle et al., 1994). Such findings challenge overly simplified conceptions of childhood SES as merely a reflection of home characteristics, underscoring that children’s socioeconomic conditions are co-constructed across multiple developmental contexts.

#### Rethinking family income as a traditional home SES Indicator

In terms of the reliability of traditional home SES indicators, there are also questions regarding family income. Many SES scholars have discussed the limitations of family income as a central SES indicator of children’s life changes and advocated for a comprehensive understanding of SES that includes the children’s own experiences Mayer (1998). argued that many models exploring how children ‘turn out’ are based exclusively on parents’ income, or parents’ income alongside additional parental characteristics (e.g., parental competence values). As a result of these studies, it was not possible to disentangle parents’ income from other parental characteristics that may impact children’s SES.

To support her argument, Mayer used five strategies to estimate the true effect of parental income. These strategies included: (a) looking at income from different sources, (b) comparing the apparent effect of parental income measured before an outcome, (c) examining the investment of parental income on child outcomes, (d) examining the association between family income and parents’ psychological well-being and parenting practices, and (e) looking at exogenous sources of variation in income that are not correlated with parental characteristics. Across five different studies, Mayer (1998) found that the effect of parental income is consistently smaller than estimates based on conventional methods (e.g., observational data).

More recently, Noble et al. (2015) examined whether family income was associated with children’s cognitive and brain development. Their findings suggested that family income was linked to critical developmental resources, such as nutrition, health care, schooling, play areas, and other physical environments, which in turn shaped children’s outcomes. Thus, they suggested that future research should disambiguate these proximal processes, focusing not just on distal indicators of SES (e.g., family income) but on the actual experiential pathways shaping children’s daily lives and development. For the purposes of our study, the Noble et al. (2015) findings reinforce our position that using family alone SES to represent childhood SES is insufficient. Their work highlights the importance of capturing additional experiences and environmental conditions, rather than replying solely on conventional SES indicators when studying childhood outcomes.

While the current study does not focus on childhood outcomes per se, it does include a broader range of SES indicators. In addition to income, we incorporate additional school-based SES indicators such as the proportion of economically disadvantaged students, free lunch, and reduced-price lunch eligibility. Together, these proximal factors are likely to provide a more comprehensive understanding of how parental mental health correlates with childhood SES indicators.

### Impacts of mental health on adult SES

Given the complexities of measuring SES, this study also investigated the relationship between childhood SES measures and parental mental illnesses. This link has been widely studied in epidemiology, typically within an explanatory framework: Social Causation and Social Selection. In their seminal study, Dohrenwend et al. (1992) reported that lower SES (educational attainment and occupation) led to major depression in adult women and antisocial personality disorder in adult men (Social Causation). Conversely they found that schizophrenia may lead to lower SES (Social Selection). Several subsequent studies have supported this framework, with income variables included in their SES constructs (e.g., Richardson et al., 2017). These studies notably highlighted financial pressure as one of the most significant factors affecting mental health (Åslund et al., 2014; Kiely et al., 2015; Richardson et al., 2017).

### Impacts of parental mental health on childhood SES

The link between parental mental health and childhood SES, shaped by multiple environments, however, is less firmly established, with a very small number of studies examining this connection. Indeed, Slominski (2010) utilized data from a 30-year, three-generation longitudinal mental health study. Using a structural equation model (SEM), Slominski (2010) found that increased maternal mental health during early childhood (30 months of age) was associated with lower SES later when children were 13 years old and 18 years old, respectively. At 30 years old, children whose mothers had increased mental health depression during early childhood were found to have lower educational attainment and lower incomes. Slominski’s (2010) study highlights the negative impacts of maternal mental health on children’s SES later in adult life.

Moreover, in another social causation and social selection study, Ritsher et al. (2001) also investigated the bidirectional relationship between parental mental health and family SES by employing mixed-model linear and logistic regression analyses. This investigation used longitudinal interview data over 17 years. Pertinent here, the investigators found no significant influence of parental depression on children’s later SES, measured via educational attainment and occupation. One possible explanation for this finding is that parental depression is not strongly associated with children’s long-term SES. Another possibility is that the previous relationship found between parental mental illness and children’s long-term SES was driven by income differences, which were not considered in the study of Ritsher et al. (2001). Both possible explanations are considered in the current association study, which focuses on the relationship at a single early time point in middle childhood.

Recognizing the challenges in comprehensively measuring SES and accurately identifying the association between parental mental health and childhood SES, we adopt an advanced design model. This model incorporates both traditional home SES measurement indicators and emerging school SES indicators, allowing for a more nuanced investigation of the complex relationship between parents’ mental health and childhood SES measures. By utilizing this advanced approach, our study aims to offer additional insights and address gaps in previous research.

### Current study

The overarching goal of this study is to determine how parental mental health is associated with childhood SES. Structural Equation Modeling (SEM) allowed for the simultaneous examination of more than one well-established latent construct of SES as an outcome. The SEM design was grounded in the Ecological Systems Theory and the Fundamental Cause Framework, with pathways in the model examining the relationships between parents’ mental health and children’s home and school SES, based on published evidence-based correlation studies.

We hypothesized that parents’ mental health would significantly correlate with measures of their children’s SES during childhood, assessed through both immediate family-level and broader school-district-level indicators. Specifically, we aim to address the following research questions:

1. Is parental mental health associated with proximal measures of school SES?
2. Is parental mental health associated with proximal measures of home SES?

## Methods

The Adolescent Brain Cognitive Development (ABCD) study is an ongoing longitudinal study designed to track the psychological and neurobiological development of children from early to late adolescence (Garavan et al., 2018). It includes a baseline group of 11,868 children aged nine and 10, along with their parents or guardians, who agreed to participate in the study for a decade beginning in 2018 (Garavan et al., 2018). Baseline assessments occurred between September 1, 2016, and August 31, 2018. Assessments included four major components: (a) interviews and questionnaires were conducted with parents for themselves and their child, (b) games and puzzles were used to assess the child’s cognitive function, (c) biological samples were collected for genetic and other tests, and (d) magnetic resonance imaging (MRI) is carried out to collect neurobiological images (Adolescent Brain Cognitive Development Study, 2024). The study consists of yearly assessments in a lab setting, which are estimated to last for six to seven hours (Garavan et al., 2018). Assessments are estimated to last for 6–7 h. Additionally, every 3–6 months, an ABCD researcher conducts a brief follow-up survey either online or by phone with the parents (Adolescent Brain Cognitive Development Study, 2024).

A significant principle of recruitment for the ABCD study is to achieve a sample that is representative of the U.S. population, reflecting its sociodemographic diversity as closely as possible. To this end, the study primarily recruited participants from public, charter, and private elementary schools (Garavan et al., 2018). In terms of school-district level data, statisticians from the University of Michigan Institute for Social Research (ISR), specializing in sampling, employed the geographic information system (GIS) software on data from the Common Core of Data (CCD), and Private School Survey (PSS), national databases from the National Center for Education Statistics (NCES). This allowed them to identify the boundaries of catchment areas for each site and the corresponding school districts (Garavan et al., 2018). This school-based recruitment is used to avoid the selection biases associated with convenience sampling methods and other non-probability sampling strategies (Baker et al., 2023). However, although this sampling strategy was designed to reflect the demographic makeup of the United States, achieving national representativeness across all variables is not automatically assured (Garavan et al., 2018). The geographic constraints of the ABCD sites, areas with neuroimaging technology and assessment expertise, resulted in a sample pool that has more participants from urban areas, leaving those from rural areas underrepresented (Garavan et al., 2018). Further, the SES of the entire ABCD cohort is skewed, with more parents who are highly educated and/or economically successful (Garavan et al., 2018). Baseline data indicate that almost 60% of parents have attained at least a bachelor’s degree. Likewise, approximately 40% of children were living in a household with a combined family income of at least $100,000 per year at the baseline. Consequently, the explanatory power of this study, on a subsample of the ABCD Cohort, on lower SES groups is likely to be inherently restricted; this is discussed further in the limitations section of this manuscript.

### Participants

The original sample included 11,868 (female 5,664; 47.74%) children average age of 9.48 years (*SD* = 0.51, MIN = 8, Max = 11). The majority of the children were racially identified as White (58.89%), Black (14.61%), Asian (3.30%), American Indian (0.46%), Other and Pacific Islander (0.54%), and Multiracial (4.03%). In the total sample, 17.22% of children were identified as Hispanic. In our analyses, we aimed to investigate the potential associations between parents’ mental health and children’s SES during childhood. As such, we limited our analyses to participants with complete SES data (details below) and available data on mother’s and father’s mental health status. This decision was made to account for the potential influence of both maternal and paternal mental health. This resulted in an analytic sample of 5,799 (female 2,694) children between the ages of 8 and 11 years. At the family level, the responding parent is predominantly female (87.24%) and White (66.96%), with a mean age of 40.63 years. Of the children, about half are females (46.46%), predominantly White (70.86%), with an average age of 9.48 years.

### Measures

This study focused on the associations between parental mental health and childhood SES. Fourteen observed variables were classified into three latent variables. These latent variables served to capture parents’ mental health, traditional childhood SES at home, and emerging childhood SES in school. Latent variables are described below in turn.

#### Parental mental health

A version of the Family History Assessment Module Screener (FHAM-S) (Rice et al., 1995) was adapted (Brown et al., 2015) and administered as a survey to the parents of children in the ABCD study. Parents’ mental health was measured using six items indicative of mental health illnesses. Three items were asked with regard to the biological mother’s mental health, and three items were asked with regard to the biological father’s mental health. Parents were asked to report the presence (coded = 1) or absence (coded = 0) of depression, mania, and symptoms of psychosis (i.e., visions of others spying/plotting). While the family history method is the most commonly used approach to assessing family mental health, it is not as sensitive as direct assessment. However, it is generally considered a reasonable approach (Andreasen et al., 1986; Rice et al., 1995).

We focused on depression, mania and psychosis because these conditions are among the most empirically studied parental mental health disorders and have well-established links to parenting difficulty, family functioning, and child developmental outcomes (e.g., Harries et al., 2023).

#### Socioeconomic status

Since SES is not a unidimensional construct but rather a multifaceted one, elements within SES latent variables can function both as causes and emergent properties at different points in life. Therefore, in this study, we attempt to separate home SES (i.e., income, parental educational attainment, and occupation) from school SES (i.e., the proportion of economically disadvantaged students and free and reduced-price lunch). Home SES is argued to represent the traditional socioeconomic foundation at the household level. In contrast, school SES focuses on the tangible circumstances that directly impact the child’s day-to-day environment. As empirical research has revealed, the contextual effects within the school settings can extend beyond the home and can significantly shape children’s learning and development outcomes (e.g., Aikens and Barbarin, 2008; Perry et al., 2022), influencing a child’s immediate experiences and potential future trajectories. By distinguishing between home SES and school SES, we aim to explore how school SES provides additional insights into childhood SES by including emergent, observable effects in school settings. This separation of linked but distinct factors helps to clarify the role of different facets of SES in the two primary environments of children (i.e., home and school), offering a more nuanced understanding of childhood SES.

Additionally, the Developmental Model of Transgenerational Transmission of Psychopathology (Hosman et al., 2009) informed our analytical decision to examine children’s developmental environments separately. This model emphasizes that parental mental health not only affects child outcomes through biological heritability but also through disruptions in the home and broader ecological contexts (e.g., school and neighborhood). Consistent with this framework, we conceptualized and modeled childhood SES using both home- and school-based measures, allowing the examination of associations between parental mental illness and inequalities in children’s developmental opportunities.

#### Traditional home SES

We incorporated parents’ occupational status and prestige, educational attainment (Likert scale: 1–5), and combined household income (see Table 1 and details below). In this study, data were included only for participants with complete SES data.

**Table 1:** Home SES (analytical sample).

Characteristic	*n*	%	Characteristic	*n*	%
Educational attainment					
Parent 1			Parent 2		
Less than High School	203	3.50%	Less than High School	355	6.12%
High School	394	6.79%	High School	685	11.81%
Some College	1,460	25.18%	Some College	1,487	25.64%
Bachelor’s Degree	1933	33.33%	Bachelor’s	1709	29.47%
Postsecondary Education	1809	31.20%	Postsecondary Education	1,563	26.95%
Occupational prestige					
Parent 1			Parent 2		
Not Applicable/NEVER had a job	169	2.91%	Not applicable/NEVER had a job	59	1.02%
Low occupation	717	12.36%	Low occupation	1,366	23.56%
Middle occupation	1901	32.78%	Middle occupation	1,529	26.37%
High occupation	3,012	51.94%	High occupation	2,845	49.06%
Family combined income					
Less than $5,000	48	0.83%			
$5,000 through $11,999	66	1.14%			
$12,000 through $15,999	54	0.93%			
$16,000 through $24,999	152	2.62%			
$25,000 through $34,999	210	3.62%			
$35,000 through $49,999	347	5.98%			
$50,000 through $74,999	764	13.17%			
$75,000 through $99,999	1,015	17.50%			
$100,000 through $199,999	2,274	39.21%			
$200,000 and greater	869	14.99%			

*N* = 5,799 (*n* for each condition).

##### Occupation

To validate employment status, the parent demographics survey initially posed a question to the survey parent (parent 1) for initial validation. Parents were asked, “Whom did you work for?” Respondents suggesting unemployment were coded as “Not Applicable/Participant has NEVER had a job.” Subsequently, employed parents were required to further specify their “Job title.” The identical procedure and questions were then applied to collect occupational data of the second parent/partner (parent 2) from the surveyed parents. In the ABCD Study®, a total of 97 parental occupations were identified.

To simplify the 97 occupational categories for analytical purposes and assign them a rank reflecting occupational prestige, given the contemporary U.S. context in which the data were collected, we used the two-step occupational Likert scale developed by Khundrakpam et al. (2020).

First, each occupation was initially broken into seven nominal classification groups: “1 = entry-level or general employees,” “2 = Machine operators and semi-skilled employees,” “3 = Skilled manual employees,” “4 = Clerical and sales workers, technicians, and owners of small businesses (<2 employees),” “5 = Administrative personnel, owners of small businesses, and minor professionals,” “6 = Business managers, proprietors of medium-sized businesses, and lesser professionals,” and “7 = Higher executives of large concerns, proprietors, and major professionals.” For example, food and beverage serving workers were coded as 1, food processing workers were coded as 2, installation, maintenance, and repair workers were coded as 3, retail sales workers were coded as 4, other personal care and service workers were coded as 5, advertising, marketing, promotions, public relations, and sales managers were coded as 6, and lawyers, judges, and related workers were coded as 7. This recategorization was conducted independently by the two authors. We achieved an inter-rater agreement on these seven occupational categories as measured by Cohen’s Kappa of 0.69 (*p* < 0.001), indicating substantial agreement between the two raters (Cohen et al., 2013). As recommended, the two raters reviewed and discussed cases where disagreements occurred and came to a consensus.

Second, we divided the seven occupational categories into four groups for ordinal classification. Specifically, the original classification group 1–3 was re-coded as the low parental occupation group (coded: low = 1), classification group 4–5 was re-coded as the middle parental occupation group (code: middle = 2), and classification group 6–7 was re-coded as the high parental occupation group (code: high = 3). Finally, those who indicated that they were not employed were added to the lowest end of the ordinal scale (coded: unemployed = 0). All groups were recoded for the analysis.

##### Combined family income

The total combined family income was acquired using one single item from the Parent Demographic Survey, reported by parent 1: “What is your TOTAL COMBINED FAMILY INCOME for the past 12 months? This should include income (before taxes and deductions) from all sources, wages, rent from properties, social security, disability and/or veteran’s benefits, unemployment benefits, workman’s compensation, help from relative[s] (includ[ing] child payments and alimony), and so on.” The response options were structured as a conventional range format: “1 = Less than $5,000; 2 = $5,000 through $11,999; 3 = $12,000 through $15,999; 4 = $16,000 through $24,999; 5 = $25,000 through $34,999; 6 = $35,000 through $49,999; 7 = $50,000 through $74,999; 8 = $75,000 through $99,999; 9 = $100,000 through $199,999; 10 = $200,000 and greater.” In the full ABCD sample, a total of 511 surveyed parents refused to answer this question, 504 responded that they did not know, and data from two respondents were missing. These individuals were excluded from the current study, which required complete SES information.

##### Educational attainment

The educational attainment was collected using two items: “What is the highest grade or level of school you have completed or the highest degree you have received?” and “What is the highest grade or level of school your partner completed or highest degree they received?” Response choices for both questions were structured in a range format: “0 = Never attended/Kindergarten only; 1 = 1st grade; 2 = 2nd grade; 3 = 3rd grade; 4 = 4th grade; 5 = 5th grade; 6 = 6th grade; 7 = 7th grade; 8 = 8th grade; 9 = 9th grade; 10 = 10th grade; 11 = 11th grade; 12 = 12th grade; 13 = High school graduate; 14 = GED or equivalent Diploma; 15 = Some college; 16 = Associate degree: Occupational; 17 = Associate degree: Academic Program; 18 = Bachelor’s degree; 19 = Master’s degree; 20 = Professional School degree (e.g., M.D.); 21 = Doctoral degree. For self-reported educational attainment, 17 participants refused to respond. For partner’s educational attainment, 17 refused to report, 44 did not know, and 2,398 responses were missing.

The original ordinal scale was restructured into categories reflecting educational levels. The new categories were: Less than High School (0–12), High School Graduate or GED (13–14), Some College (15), Associate’s or Bachelor’s Degree (16–18), and Postsecondary Education (19–21).

#### School SES

School SES is designed to reflect broader socioeconomic conditions that are associated with but not subsumed by Home SES. All three indicators within the School SES measurement were sourced from the Stanford Education Data Archive (SEDA), one of the most comprehensive and nationally representative longitudinal datasets integrated within the ABCD Study®. SEDA offers geographic data at the school district level, derived from federally mandated assessments (Fahle et al., 2017). Within the construct of School SES, the specific indicators are the proportion of students eligible to receive reduced lunch, the proportion of students eligible to receive free lunch, and the proportion of students considered economically disadvantaged.

The proportion of students eligible for reduced lunch, reported by ABCD participants’ schools served as a proxy for school SES (Garavan et al., 2018). This measure, referred to as ‘Proportion Reduced Lunch Eligible,’ was calculated as a weighted average over the years 2009–2018 using data from the Common Core of Data (CCD). Similarly, the proportion of students eligible for free lunch, another key indicator reported by ABCD participant schools, served as a proxy for school SES (Garavan et al., 2018). This measure, referred to as the “Proportion Free Lunch Eligibility,” was also calculated as a weighted average over the years 2009–2018 using CCD data. Lastly, the proportion of economically disadvantaged students, referred to as “Proportion Economically Disadvantaged,” was calculated as a weighted average over the 2009–1,018 period. This metric, based on data from EdFacts, varies by state definitions but generally captures the percentage of students who face significant economic hardship (Garavan et al., 2018).

In the Results section, we reported the statistics for the three indicators used to measure school-district-level SES. Detailed descriptions of the measurement approaches for these items and federal statistics can be found in Table 2.

**Table 2:** School SES (analytical sample).

Variables	*M*	*SD*	Skewness
Proportion economically disadvantaged (Ed Facts)^a^	0.40%	0.26	0.63
Proportion free lunch eligible (CCD)	0.32%	0.24	0.84
Proportion-reduced lunch eligible (CCD)	0.06%	0.4	0.94

*N* = 5,799 (*n* for each condition). This table presents school district-level SES variables based on a 2009–2018 weighted average. The data was refined by statisticians from the University of Michigan’s Institute for Social Research (IRS) using the geographic information system (GIS) software, alongside the Common Core of Data (CCD) and Private School Survey (PSS) national databases from the National Center for Education Statistics (NCES). This process involved converting catchment area boundaries into corresponding school districts to accurately reflect the student populations served within those areas (Garavan et al., 2018). ^a^Economically disadvantaged is defined by states.

### Analytic plan

Prior to the data analyses, IBM SPSS Statistics 29.0.2.0 (20) was used to prepare the data. A Missing Values Analysis indicated that Little’s (1988) test of Missing Completely at Random (MCAR) was conducted on the full sample. All observed variables, namely sociodemographic variables (i.e., age, race, and gender) of both parents and children, SES indicators, and parents’ mental health variables, were included in the test. Little’s test was not significant, χ^2^ = 2.19, DF = 2, *p* = 0.33. Results indicated that data were missing completely at random, suggesting no systematic differences between the participants with missing data and those with complete data. As such, listwise deletion was used to create the analytic sample for statistical analyses, which included SES information for two parents.

Multivariate outliers were detected using Cook’s distance (Cook, 1977), which represents a statistic that combines standardized residuals with leveraged values to spot influential variables. Any observations with a Cook’s distance greater than one standard deviation from the mean are recommended for elimination (Cohen et al., 2013). No multivariate outliers were found in any of the continuous variables (i.e., proportion reduced lunch eligible, proportion free lunch eligibility, and proportion economically disadvantaged) included in the analyses, resulting in a final sample of 5,799 individuals.

R version 4.3.1 (2023-06-16) was used for all structural equation model (SEM) analyses. A Confirmatory Factor Analysis (CFA) was used to examine the factor structure of parental mental health, child School SES, and Home SES. The model contains three latent constructs: Parent Mental Health, School SES, and Home SES. Parent Mental Health included six measured variables: (a) father depression, (b) mother depression, (c) father mania, (d) mother mania, (e) father psychosis, and (f) mother psychosis. School SES included three measured variables: (a) proportion of economically disadvantaged students, (b) free lunch, and (c) reduced-price lunch. Home SES included five measured variables: (a) father’s educational attainment, (b) mother’s educational attainment, (c) household income, (d) father’s occupational prestige, and (e) mother’s occupational prestige.

A Latent Variable Path Analysis (LVPA) was conducted to address our primary research questions. Specifically, the LVPA estimated the associations between parents’ mental health and the children’s School and Home SES. A dampened weighted least squares (DWLS) estimator was used in the analysis. The DWLS is a robust estimator that can provide more accurate factor loadings and structural coefficients for structural equation models with categorical variables, especially under conditions of non-normal data distribution, than maximum likelihood-based estimators (Bandalos, 2014; Li, 2016, 2021). The following model goodness-of-fit tests were reported for the LVPA model: root mean square error of approximation (RMSEA) (Browne and Cudeck, 1993), with 90% confidence intervals (CIs) and comparative fit index (CFI) (Bentler, 1990). To indicate a good model fit, models were evaluated against the following guidelines: the RMSEA estimate should be lower than 0.05 (Hu and Bentler, 1999), CFI should be greater than 0.90 (Bentler and Bonett, 1980), and SRMR should be less an 0.05 (Byrne, 1998).

## Results

### Descriptive statistics

Table 3 provides correlations for all the observed variables included in the analytical models. All variables within the Home SES construct were significantly correlated (*p* < 0.01). The same is true of the variables within the School SES construct (*p* < 0.01). Notably, the proportion of economically disadvantaged students and the free lunch variables within the School SES were highly correlated (*r* = 0.98). To monitor collinearity and redundancy to ensure that the models remain robust and interpretable, variance inflation factors (VIFs) were checked during the model estimation process through regression analysis and found to be within the acceptable ranges (1.02–1.07). A VIF value of 10 has been widely adopted as a criterion for significant collinearity, while 5 may suggest collinearity (Hair et al., 2006; O’Brien, 2007; Menard, 2001). On one hand, including both measures remains important for policy and intervention analysis. The proportion of economically disadvantaged students, defined by states, reflects a broader measure of financial hardship, while the free lunch variable provides a direct measure of poverty-related assistance in schools. On the other hand, strong correlations among observed variables within a latent construct enhance reliability, validity, and accurate estimation. Significant correlations among all variables within parental mental health construct (*p* < 0.01).

**Table 3:** Correlations matrix of observed variables.

Variable	1	2	3	4	5	6	7	8	9	10	11	12	13
1. Parent 1 education													
2. Parent 2 education	0.60**												
3. Combined income	0.59**	0.55**											
4. Parent 1 occupation	0.44**	0.31**	0.38**										
5. Parent 2 occupation	0.40**	0.51**	0.44**	0.21**									
6. Disadvantage proportion	−0.43**	−0.45**	−0.54**	−0.25**	−0.34**								
7. Free lunch	−0.43**	−0.44**	−0.54**	−0.25**	−0.34**	0.98**							
8. Reduced-price lunch	−0.23**	−0.25**	−0.26**	−0.13**	−0.18**	0.56**	0.47**						
9. Father depression	0.00	0.01	−0.04**	0.02	−0.01	0.00	0.01	−0.00					
10. Mother depression	−0.02	−0.01	−0.03*	−0.00	−0.01	0.01	0.01	0.01	0.21**				
11. Father mania	−0.02	−0.01	−0.04**	0.00	−0.03	0.03*	0.03*	0.03*	0.24**	0.06**			
12. Mother mania	−0.05**	−0.02	−0.06**	−0.05**	−0.02	0.05**	0.05**	0.03	0.09**	0.19**	0.10**		
13. Father VS	−0.06**	−0.06**	−0.10**	−0.04**	−0.04**	0.07**	0.08**	0.02	0.17**	0.07**	0.29**	0.11**	
14. Mother VS	−0.01	−0.02	−0.05**	−0.00	−0.01	0.05**	0.06**	0.01	0.07**	0.10**	0.04**	0.13**	0.12**

*N* = 5,799. The 95% confidence interval is a plausible range of population correlations that could have caused the sample correlation (Cumming, 2014). VS = visions of others spying/plotting problems. * Indicates *p* < 0.05. ** Indicates *p* < 0.01.

The analytical sample size is 5,799. As shown in Table 4, the vast majority of primary parents (coded as Parent 1) who participated in the ABCD study and responded to questions regarding themselves, their partners, and their children (87.24%) identified as female. Most of these primary respondents were White (66.96%). The average age of these parents was 40.63 years (*SD* = 5.92). Regarding the children, 46.46% were female, and the majority identified as White (70.86%). The average age of the children was 9.48 years (*SD* = 0.51). Although this analysis focuses on two parents, the descriptive statistics presented here reflect data from the primary respondent parent.

**Table 4:** Sociodemographic characteristics (analytical sample).

Characteristics	*n*	%/Mean (SD)	Characteristic	*n*	%
Parent gender identity – Female	5,059	87.24%	Child gender identity – female	2,694	46.46%
Transgender and Other Identities	12	0.21%			
Parent race			Child race		
American Indian	17	0.29%	American Indian	15	0.26%
Asian	117	2.02%	Asian	214	3.69%
Black	327	5.64%	Black	327	5.64%
White	3,825	66.96%	White	4,109	70.86%
Other and Pacific Islander	34	0.59%	Other and Pacific Islander	24	0.41%
Multiracial (Non-Hispanic)	576	9.93%	Multiracial (Non-Hispanic)	192	3.31%
Hispanic	885	15.26%	Hispanic	880	15.18%
Parent age		40.63 (5.92)	Child age		9.48 (0.51)
Under 30	164	2.83%	8	3	0.05%
30–34	705	12.16%	9	3,019	52.06%
35–39	1,569	27.06%	10	2,755	47.55%
40–44	1913	32.99%	11	17	0.29%
45–49	1,077	18.57%			
50–59	324	5.59%			
60 or older	26	0.45%			

*N* = 5,799 (*n* for each condition). ^a^ Reflects the number and percentage of participants answering “yes” to this question. ^b^ Other Identities: Gender Queer/Different Other Identity.

Table 1 illustrates the traditional Home SES characteristics of the analytical sample, focusing on educational attainment, occupational prestige, and family combined income. For Parent 1, the majority held a Bachelor’s degree (33.33%) or higher levels of postsecondary education (31.20%). Parent 2 shows a similar pattern. In terms of occupational prestige, over half of Parent 1 (51.94%) and nearly half of Parent 2 (49.06%) held high-occupation jobs. Regarding family income, most families fell within the middle-to-upper income ranges, with 39.21% earning between $100,000 and $199,999 annually. Again, we note that the analytic sample was representative of the full sample, with both income and educational attainment positively skewed. The ABCD study aims to be representative of the diverse U. S. population, with efforts to include participants from a wide range of socioeconomic backgrounds, including SES. However, the full sample exhibited a positive skew in SES, with higher income and educational attainment more prevalent. To account for this skewness, statistical techniques were applied to the analytical sample (a discussion of the use of the DWLS estimator can be found in the previous Analytical Plan section).

Table 2 presents the emerging School SES characteristics of the analytical sample, focusing on school district-level SES variables based on a 2009–2018 weighted average. The proportion of economically disadvantaged students, as defined by states, was negatively skewed at 0.40 (*SD* = 0.26), with a skewness of 0.63 and a kurtosis of −0.67. The proportion of students eligible for free lunch was negatively skewed at 0.32 (*SD* = 0.24), with a skewness of 0.84 and kurtosis of −0.32. The proportion of students eligible for reduced-price lunch was positively skewed 0.06 (*SD* = 0.04), with a skewness of 0.94 and kurtosis of 2.50.

Table 5 presents the mental health status of parents in the analytical sample. It shows that 19.68% of mothers and 12.71% of fathers were experiencing depression, 1.41% of mothers and 1.55% of fathers were experiencing mania, and <1% (0.69%) of mothers and <1% (0.41%) of fathers were experiencing visions of others spying or plotting against them.

**Table 5:** Parents’ mental health (analytical sample).

Characteristic	*n*	%	Characteristic	*n*	%
Depression					
Mother	1,141	19.68%	Father	737	12.71%
Mania					
Mother	82	1.41%	Father	90	1.55%
Visions of others spying/plotting problem					
Mother	40	0.69%	Father	24	0.41%

*N* = 5,799 (*n* for each condition).

### Confirmatory factor analysis

**Figure 1:**
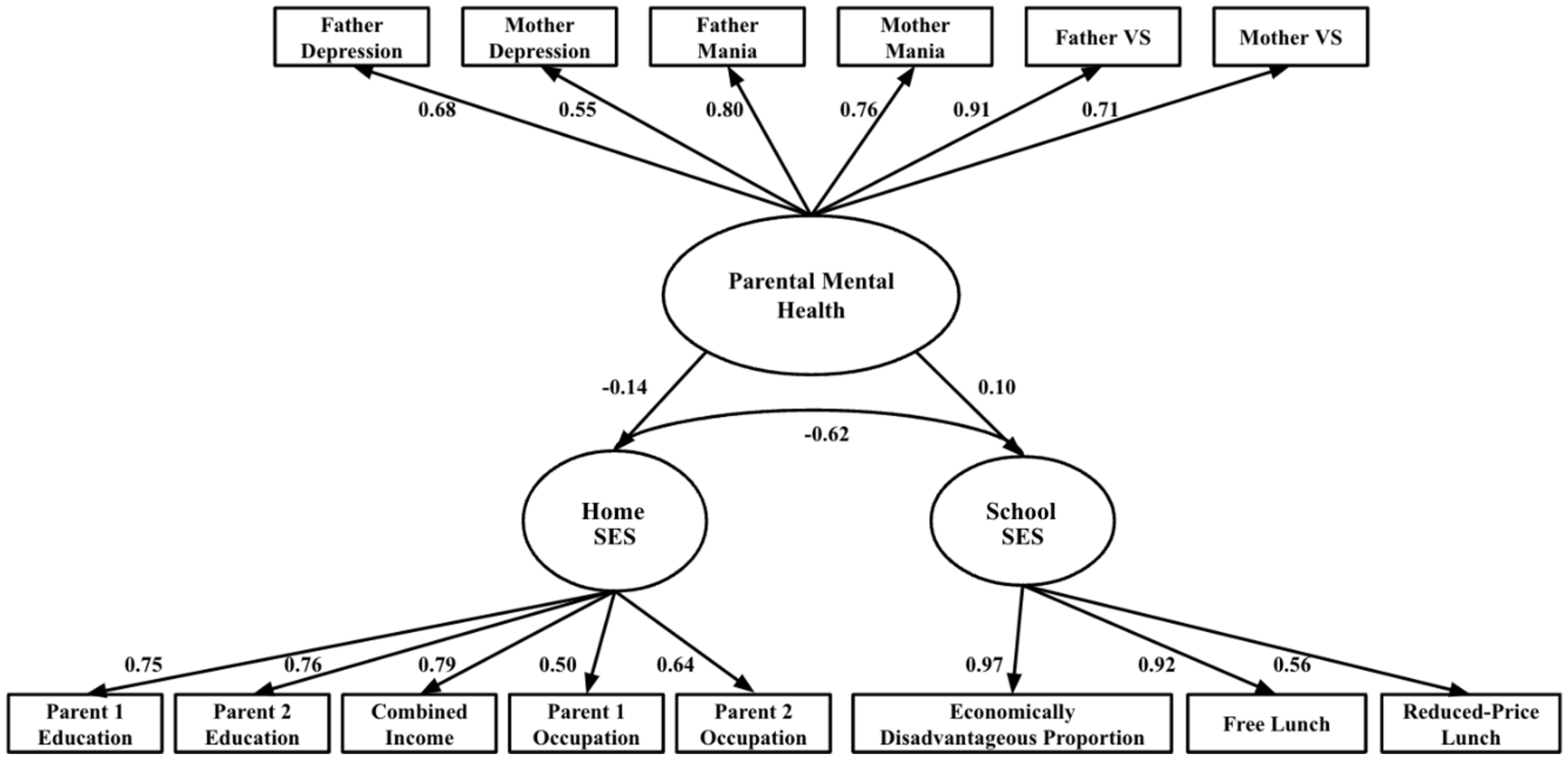
Confirmatory factor analysis of parental mental health, home SES, and school SES. The fit of the model was excellent, with χ^2^ (74) = 722.29, *p* < 0.001; CFI = 0.99; RMSEA = 0.04, 90% CI [0.04, 0.06]; SRMR = 0.09. All standardized loadings for the observed variables on their latent factors were significant (*p* < 0.001). VS = visions of others spying/plotting problems. All coefficients are standardized. All paths are significant at *p* < 0.001.

A Confirmatory factor analysis CFA was conducted to test the measurement model. The fit of the model was assessed and found to be excellent, with χ2 (74) = 722.29, *p* < 0.001; CFI = 0.99; RMSEA = 0.04, 90% CI [0.04, 0.06]; SRMR = 0.09. All standardized loadings for the observed variables on their latent factors were significant (*p* < 0.001), as in Figure 1. Thus, these results indicate a well-fitting model with all observed variables significantly loading on their construct, supporting the validity of the measurement model. Three latent constructs were established as (1) Parental Mental Health, (2) Home SES, and (3) School SES.

### Latent variable path analysis (LVPA)

The LVPA model was then conducted to examine the relations among latent constructs. The model provided a very good fit to the data with χ2 (74) = 722.29, *p* < 0.001; CFI = 0.99; RMSEA = 0.04, 90% CI [0.04, 0.06]; SRMR = 0.09, despite the significant structural paths observed in the LVPA model. This phenomenon is most likely attributed to the identified strong measurement model and the large analytical sample sizes (*N* = 5,799), which provide stability and robustness to model fit measures (Kline, 2016). The structural paths LVPA model revealed significant relations between Parental Mental Health and both School and Home SES. Specifically, Parental Mental Health was positively associated with School SES (*β* = 0.10, *p* < 0.001), indicating that affected parents’ mental health was linked to children attending schools with higher proportions of economically disadvantaged students and greater eligibility for free/reduced-price meal programs. Conversely, Parental Mental Health was negatively associated with Home SES (*β* = −0.14, *p* < 0.001), indicating that affected parents’ mental health was associated with lower socioeconomic conditions at home, as indicated by lower parental educational attainment, occupational prestige, and family income.

## Discussion

Extensive global research demonstrates that socioeconomic disadvantage and social inequalities have a profound influence on children’s health and developmental trajectories (e.g., Brooks-Gunn and Duncan, 1997; Marmot and Bell, 2012), and evidence further shows that poverty and parental mental illness have compounding effects (e.g., Adjei et al., 2024). In the United States, more than one in every 14 children lives with a parent who has a mental illness (Wolicki et al., 2021), and this pattern reflects a broader global reality in which approximately one billion people experience mental illness (World Health Organization, 2025). These prevalence statistics underscore the urgent need to explore the importance of examining how the mental health of parents affects their children, especially in terms of providing economic and social support. Children growing up in households where adults struggle with mental health difficulties are more likely to experience poor general health, increased poverty, neglect, and other adverse childhood experiences (Murthy, 2024). As such, this study aimed to determine whether parental mental health was associated with childhood SES using a more comprehensive measure of childhood SES that includes both the home and school environments. Below, we discuss these findings in the context of the broader literature.

First, we found a significant positive association between parental mental illnesses and School SES, indicating that children of affected parents were likely to experience economic disadvantage outside the home. This is consistent with prior evidence. For example, Reupert and Maybery (2007) found that the economic strain associated with parents’ mental illnesses can significantly limit children’s access to basic needs. These economic challenges often lead to increased eligibility for school-based financial support, such as free and reduced-price meals, and a higher likelihood of attending schools in economically disadvantaged areas. These findings suggest that the school environment, where children spend a substantial portion of their time and engage in crucial developmental activities, is adversely affected. Further, the economic constraints of these environments can limit access to quality educational resources, extracurricular opportunities, and social networks (e.g., Sirin, 2005; Reardon, 2018; Mullis et al., 2016).

Second, we found a negative association between parental mental health and Home SES, indicating that children of affected parents were more likely to experience economic disadvantage within the home. This is also consistent with prior evidence. For example, Smith (2004) found that poverty, severe economic deprivation, and social isolation are often associated with parents’ mental illness, exacerbating the challenges faced by children in such environments. Children growing up in such home environments may lack access to vital educational and developmental opportunities, impeding their academic achievement and career prospects (Farahati et al., 2003; Sherman and Hooker, 2018; Tabak et al., 2016). This can perpetuate low socioeconomic status across generations, as the lack of resources and support in early life limits a child’s ability to improve their socioeconomic position in adulthood (e.g., Conger et al., 2010; Duncan et al., 2010; Heckman, 2006). This indicates not only early disadvantaged family resources for the child but also a potential long-term cycle of disadvantage that can limit the child’s upward social mobility (e.g., McLoyd, 1998; Shonkoff and Garner, 2012). Further, previous studies on later life SES outcomes report far-reaching influences of parental mental health on children’s future socioeconomic trajectories during their own adulthood (e.g., Slominski, 2010; Slominski et al., 2011).

This study has two notable strengths. First, it advances conceptual and measurement clarity by challenging the conventional assumption that childhood SES can be fully represented by parental characteristic-based SES. Children’s developmental opportunities are shaped not only by their families’ socioeconomic circumstances but also by the broader social environments in which they learn and grow. Through a thorough review of SES literature, this study elucidates how children’s socioeconomic conditions are co-constructed across home and school contexts, where different resources, opportunities, and constraints exist. By capturing the multiple settings that collectively determine children’s access to opportunities for learning, growth, and mobility, this study provides a more ecologically representative measurement of childhood SES.

Second, this study adopts a broader and more inclusive conceptualization parental mental illness. Our review of the parental mental illness literature revealed that many empirical studies have focused solely on maternal depression, often under the umbrella of “parental mental illness.” Accordingly, they overlook other types of mental illness and the influence of fathers. While the focused examination of maternal depression has provided significant insights in the field, this scope may limit understanding of how other forms of parental mental illness shape children’s socioeconomic environments. Guided by Hosman and colleagues’ (2009) two-parent model, which specifically underscores the significance of including two parents and a wider range of mental illness, this study examines depression, mania, and psychosis for both mothers and fathers. This inclusion increases the ecological validity of the analysis and offers a more comprehensive understanding of how parental mental illness, in its varied forms, relates to children’s socioeconomic conditions.

### Limitations

This study benefits from a large sample size and the comprehensive examination of childhood SES proximal measures. However, three components of this study are worth noting as potential limitations. First, the cross-sectional nature of the data did not allow for the examination of changes in either mental health or socioeconomic status over time or generations. More importantly, the study’s correlational design did not allow for establishing a causal relationship between parental mental health and SES. Reverse causality also could not be ruled out, as it is unclear whether the observed associations reflected the true direction of potential causation, as previous literature has shown that SES can impact mental health just as mental health can influence SES (e.g., Andreu-Bernabeu et al., 2023; Jiménez-Solomon et al., 2024). This relationship between mental health and SES is complicated and bidirectional. On one hand, poor mental health can negatively influence individuals’ ability to work, gain income, or access essential social resources, leading to lower SES. On the other hand, low SES can contribute to significant financial stress, limited access to health services, and other factors that can worsen mental health. Thus, while poor mental health may be linked to lower SES in some cases, lower SES can also be related to poor mental health, suggesting a reciprocal relationship.

Second, the analytical sample in this study includes only observations where responses were available for two parents. This was chosen to thoroughly examine the association of both maternal and parental mental health and socioeconomic status. In addition, information about households was not available. While the marital status variable was collected, it did not further provide detailed information about living arrangements, such as whether single parents are cohabiting in the same residence. Therefore, we cannot draw conclusions based on the assumption of single or two-parent households. Future researchers may wish to consider various family types.

Third, data sourced from the ABCD study were essentially non-normally distributed due to its recruitment process. This resulted in a sample that is skewed towards a middle-high income, middle-high education, and/or middle-high occupational prestige. Thus, the current study may not accurately detect nuanced differences among the most economically disadvantaged groups or represent the general population. Finally, it is important to acknowledge that our data did not include measures of neighborhood SES, which represents one of the proximal environments in Bronfenbrenner (1979, 1994). Prior research indicates that living in a dangerous neighborhood can negatively affect children’s health and developmental outcomes (e.g., Minh et al., 2017), suggesting that neighborhood SES could be an additional important component of modeling childhood SES.

### Implications

This study contributes to a specific understanding of how parental mental health relates to children’s socioeconomic conditions, which is not a child outcome per se, yet a critical contextual pathway in development. Aligning with Ecological Systems Theory (Bronfenbrenner, 1979), the findings show that parental mental illness is not only an individual-level issue but one that influences multiple layers of a child’s developmental ecology. Based on our findings, parents with mental illness tend to have lower education, lower income, and work in lower-status or lower-skilled occupations, which is associated with reduced home SES. At the same time, their children are more likely to attend schools with higher concentrations of economic disadvantage, which indicates that the effects of parental mental illness are likely to extend beyond the immediate small home setting into broader social institutions.

These findings on the cross-contextual socioeconomic consequences for children have important implications for policymaking and practice design. First, current policies and practices rarely address parental mental illness directly (Nicolson et al., 2001), compared to the wide range of supports for children (e.g., Head Start, school-community hub models). When interventions for parents do exist, they tend to focus solely on treating individual symptoms while overlooking the socioeconomic constraints intertwined with mental illness (Nicolson et al., 2001). Guided by the Fundamental Cause Framework (Hayward and Gorman, 2004), which underscores that health disparities limit access to socioeconomic resources and reinforce inequality, effective interventions must address both mental health treatment and the structural determinants that limit families’ access to resources. This could include: (1) accessible high-quality mental health care for parents, and (2) socioeconomic support, such as income or public assistance to meet basic needs, employment and job training programs, and educational accommodations to improve life opportunities. At the same time, it is critical to provide support for children who are affected by parental mental illness.

School-based mental health services, academic supports, enrichment programs, and community resource coordination serve as critical buffers against the negative effects of parental mental illness (Arenson et al., 2019; Cappella et al., 2008). However, because these children are more likely to enroll in schools with higher concentrations of economic disadvantage, these supports cannot rely solely on school capacity. Policies must allocate additional funding and personnel to high-need schools and facilitate cross-systems collaboration between education, public health, and social services to ensure these children’s equitable access to comprehensive support.

Ultimately, this study highlights that parental mental illness is not merely an individual health issue or a private family difficulty, but a potential hidden structural inequality that may costs children critical life opportunities. Addressing this less studied inequality effectively will require further research that examines how parental mental illness interacts with social identities, as well as systems of inequality to collectively shape children’s early educational and social environment, where learning opportunities, skill development, and future socioeconomic mobility emerge. Such research, along with macro-level public policies, is essential to support affected parents and children. Reducing this hidden inequality in family mental health fosters socioeconomic well-being across generations.

### Future directions

These findings underscore the need to investigate the mechanisms and conditions through which parental mental health affects children’s socioeconomic environments. Future research could also investigate how timing, chronicity, and severity of parental mental illness differently affect children’s SES across development, as these dimensions rarely considered in existing literature. Additionally, longitudinal or intergenerational study designs are particularly needed to determine whether parental mental health contributes to the long-term reproduction of socioeconomic inequalities across education, income, family formation, and employment domains. Without such designs, we risk remaining uncertain whether parental mental illness is a temporary childhood adversity or a key driver of long-term inequality.

Methodologically, future research could benefit from adopting advanced techniques and integrating an intersectional lens. An intersectional approach would allow researchers to examine identities and disadvantages (e.g., gender × SES × parental mental health) as well as social institutions and structures (e.g., family, school, education systems, policy environments) jointly, rather than separately. Moreover, researchers may wish to consider mediators (e.g., parenting practices, stigma, access to resources) and moderators (e.g., social support, community services, policy protections) to identify potential protective factors. Without advanced and intervention-focused study designs, the field risks continuing to describe disadvantages rather than identifying the most vulnerable populations and effective interventions strategies.

## Data Availability

The data used in this study come from the Adolescent Brain Cognitive Development (ABCD) Study®. The data are available through the NIMH Data Archive (NDA) at https://nda.nih.gov/abcd/. Access requires user registration and agreement to the NDA Data Use Terms. Further inquiries regarding the data can be directed to the corresponding author.
